# Long intergenic non-protein coding RNA 324 prevents breast cancer progression by modulating miR-10b-5p

**DOI:** 10.18632/aging.103021

**Published:** 2020-04-18

**Authors:** Bo Wang, Yangyang Zhang, Haitian Zhang, Faquan Lin, Qixin Tan, Qinghong Qin, Wei Bao, Yi Liu, Jiaying Xie, Qiyan Zeng

**Affiliations:** 1 Department of Biochemistry and Molecular Biology, Guangxi Medical University, Nanning 530021, Guangxi, P.R. China; 2 Department of Gastrointestinal and Gland Surgery, The First Affiliated Hospital of Guangxi Medical University, Nanning 530021, Guangxi, P.R. China; 3 Department of Clinical Laboratory, The First Affiliated Hospital of Guangxi Medical University, Nanning 530021, Guangxi, P.R. China; 4 Department of Breast Surgery, Guangxi Medical University Tumor Hospital, Nanning 530021, Guangxi, P.R. China

**Keywords:** breast cancer, LINC00324, miR-10b-5p, E-cadherin, ceRNA

## Abstract

Mounting evidence suggests that long noncoding RNAs serve as specific biomarkers and potent modulators of multiple cancers. Long intergenic non-protein coding RNA 324 (LINC00324) is ubiquitously expressed in various tissues, including breast cancer. The biological function of LINC00324 in the development and progression of breast cancer remains unknown. Here, we fully elucidate the relation between LINC00324 expression and breast cancer, and suggest a potential mechanism of action. We found that decreased expression of LINC00324 was dramatically correlated with malignancy of breast cancer, both in breast cancer tissues and in cell lines. Overexpression of LINC00324 in MDA-MB-231 cells resulted in a decrease in cell proliferation, invasion, and migration, while increasing cells apoptosis. On the other hand, loss-of-function experiments indicated that deficiency of LINC00324 promoted malignant phenotypes in breast cancer cells. Mechanically, we found that LINC00324 is mainly distributed in the cytoplasm, fostering the expression of E-cadherin by sponging miR-10b-5p. Taken together, these findings suggest that LINC00324 plays a critical role in breast cancer progression by directly interacting with miR-10b-5p. LINC00324 can thus potentially act as an early diagnostic marker and a novel therapeutic agent for breast cancer.

## INTRODUCTION

Breast cancer remains the most common cause of cancer-related death in female patients, although mortality rates have decreased in most developed countries. According to published statistics, morbidity of breast cancer has surpassed uterine cancer, leapping to the top of female malignant tumors list in some cities of China [1]. According to distinct gene expression, breast cancer can be divided into at least five subtypes, including Luminal A, Luminal B, HER-2+, normal-like, and basal-like breast cancer [2, 3]. Highly powerful clinical treatment modalities for breast cancer, including chemoradiotherapy and surgical resection, have greatly improved over the past decade. And yet, the overall recurrence and metastasis rates remain high. Elucidating the molecular mechanism of and devising efficient treatment strategies for breast cancer are highly desirable.

Long non-coding RNA (lncRNA), a heterogeneous group of non-protein coding RNA, is transcribed from intergenic or intragenic regions and lacks conserved open reading frame [4]. Cumulative evidence strongly suggests that lncRNAs participate in numerous biological processes, including cell proliferation, cell differentiation, apoptosis, and tumorigenesis [5, 6]. The ectopic expression of lncRNA is closely related to diverse types of cancer, including breast cancer [7]. For instance, decreased expression of NF-κB interacting lncRNA (NKILA) is correlated with breast cancer metastasis through regulation of NF-κB signaling in breast cancer patient [8]. LncRNA metastasis-associated lung adenocarcinoma transcript 1 (MALAT1) is highly abundant in normal mammary tissues, and its expression level is inversely correlated with breast cancer progression and metastasis [9]. Moreover, overexpression of the long intergenic non-coding RNA for kinase activation (LINK-A) decreases immunosurveillance through downregulation of antigen presentation by cancer cell, thereby initiating metastatic mammary gland tumors [10]. It is therefore clear that relevant lncRNA might be a potent target for breast cancer treatment. The potential mechanism of these molecules remains to be further explored.

LncRNA influences cancer tumorigenesis and progression through interaction with downstream signaling molecules. Studies have demonstrated that lncRNA interacts with miRNA through a pattern of competitive endogenous RNA (ceRNA). LncRNA directly binds and antagonizes the inhibiting function of miRNAs against specific target molecules [11]. Furthermore, several studies have shown that lncRNA modulates breast cancer-related biological activities such as metastasis, proliferation, and the epithelial–mesenchymal transition (EMT) processes, by restraining miRNA function [12–14].

Herein, we assessed the expression of LINC00324 in breast cancer, and we report, for the first time, a potential mechanism associated with the progression of the disease. Based on our data, gain- or loss-of-function approaches indicated that LINC00324 is a suppressor of breast cancer-related proliferation, migration, invasion, and EMT. Mechanistically, LINC00324 inhibits the EMT process in breast cancer by sponging miR-10b-5p and, as a result, restraining the progression of breast cancer. Given that miRNA may be a target for anti-cancer therapy [15], overexpression of LINC00324 could be considered as a new therapeutic approach for restricting breast cancer activities. It can also act as a biomarker for breast cancer classification.

## RESULTS

### In silico analysis of LINC00324 expression in breast cancer

**Figure 1:**
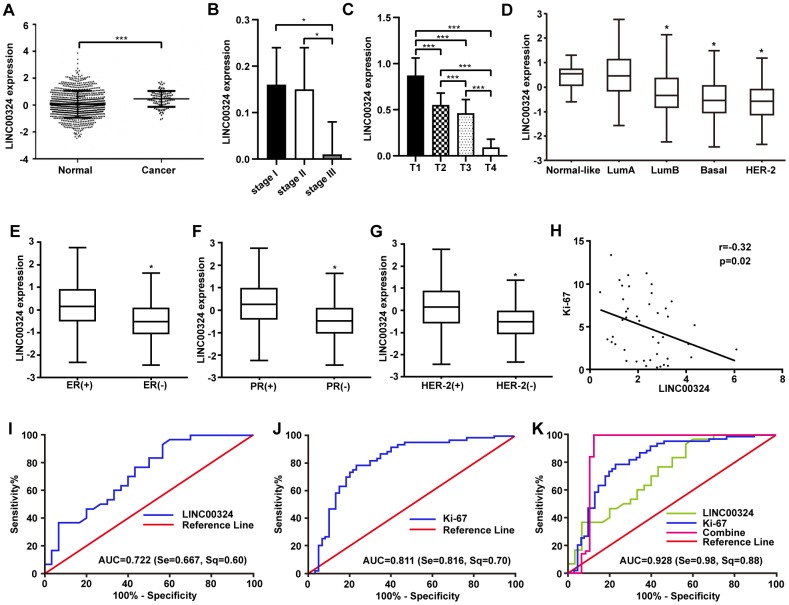
**Highly expressed LINC00324 that is involved in breast cancer was identified by bioinformatics prediction.** (**A**) Relative expression of LINC00324 in normal and breast cancer tissue. (**B**, **C**) Relative expression of LINC00324 in different clinical stages of breast cancer. (**D**) Relative expression of LINC00324 in five molecular subtypes of breast cancer. (**E**) Relative expression of LINC00324 in estrogen receptor (ER)-positive and -negative breast cancer. (**F**) LINC00324 expression in progesterone receptor (PR)-positive and -negative breast cancer. (**G**) LINC00324 expression in HER-2-positive and -negative breast cancer. (**H**) Pearson’s correlation curve showing the negative correlation between the expression of LINC00324 and Ki-67 in breast cancer. (**I**–**K**) ROC curve for breast cancer diagnostic value of LINC00324 and Ki-6. All data are shown as means ± SEM. * *P* < 0.05, *** *P* < 0.001.

To determine the expression of LINC00324 and the relative prognosis in breast cancer, we collected and assessed data on 960 patients with breast cancer via TCGA (https://cancergenome.nih.gov/) and GEO (https://www.ncbi.nlm.nih.gov/geoprofiles/) databases. The results showed a decreased expression of LINC00324 in breast cancer tissues compared with adjacent normal tissues (Figure 1A). We further investigated the relationship between LINC00324 expression and clinicopathologic features of breast cancer. Data analysis suggested that the expression of LINC00324 has a potent correlation with clinical stage and TNM stage in breast cancer patients (Supplementary Table 1). Compared with clinical stages I and II, the expression of LINC00324 was dramatically lower in stage III (Figure 1B). Downregulation of LINC00324 expression was observed in the T stage of TNM breast cancer classification and was closely related to the increase in tumor size and volume (Figure 1C). To further clarify the expression of LINC00324 based on the classification of breast cancer, decreased expression of LINC00324 was detected in Luminal B, Basal-like, and HER-2 breast cancers as well as ER, PR and Her-2 negative breast cancers, based on analysis of data from the TCGA and GEO databases (Figure 1D-1G). In order to assess the possible role of LINC00324 in breast cancer as a valuable clinical diagnostic biomarker and therapeutic target, the correlation and ROC curve analysis of Ki-67 and LINC00324 were performed. Expression level of LINC00324 was significantly negatively correlated with Ki-67 (Figure 1H). As showed in Figure 1I, the area under the ROC curve (AUC) of LINC00324 in breast cancer was 0.722. The sensitivity and specificity were 0.667 and 0.6, respectively. The AUC of Ki-67 was 0.811, and the sensitivity and specificity were 0.816 and 0.7, respectively (Figure 1J). We further analyzed the value of the combined diagnosis of LINC00324 and Ki-67 together and found that the AUC was 0.902 (sensitivity = 0.980, specificity = 0.880; Figure 1K). These results indicate that the sensitivity and specificity of LINC00324 and Ki-67 combined diagnosis were significantly higher than any of them alone, and that LINC00324 can be expected to serve as a valuable pathological diagnosis and prognosis evaluation marker for breast cancer.

### Tissue-based assessment indicate association between LINC00324 downregulation and poor prognosis of breast cancer patients

**Figure 2:**
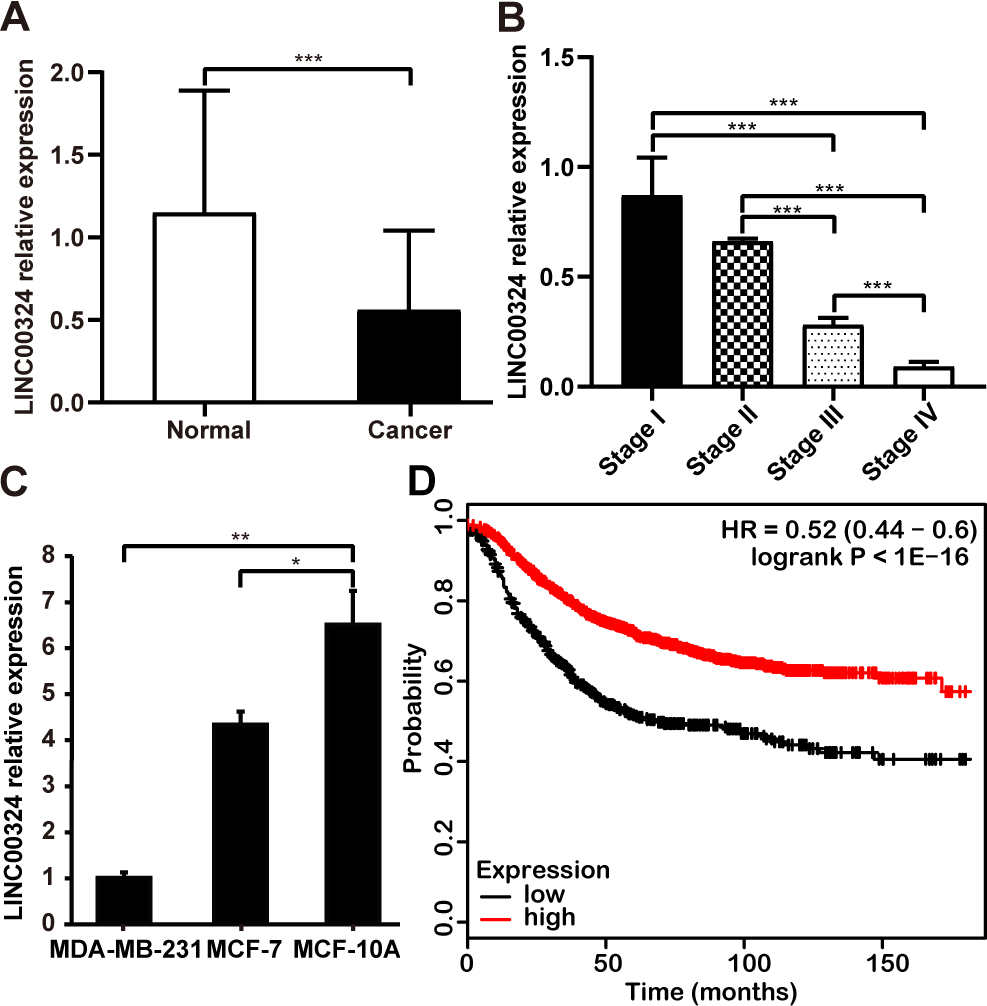
**Downregulation of LINC00034 expression predicts worse prognosis for patients with breast cancer.** (**A**) qRT-PCR analysis of LINC00324 expression in breast cancer tissues and paired adjecent normal tissues after normalization to GAPDH. (**B**) qRT-PCR analysis of LINC00324 expression in different TNM stages after normalization to GAPDH. (**C**) qRT-PCR analysis of LINC00324 expression in MDA-MB-231, MCF-7, and MCF-10A cells, after normalization to GAPDH. (**D**) Kaplan–Meier analysis for overall survival based on low and high LINC00324 expression levels (the KM Plotter database). All data are shown as means ± SEM. * *P* < 0.05, ** *P* < 0.01, *** *P* < 0.001. Data are from three independent experiments (**C**).

To further verify the expression and clinical significance of LINC00324 in breast cancer, tissue samples derived from breast cancer patients were studied and the correlation between LINC00324 expression level and clinicopathologic features of breast cancer was calculated. Level of LINC00324 was significantly lower in breast cancer tissues compared to adjacent normal tissues (Figure 2A). As the level of clinical staging increased, the expression level of LINC00324 gradually decreased in the breast cancer tissue samples (Figure 2B). Furthermore, we explored the expression of LINC00324 in normal (MCF-10A) and breast cancer (MDA-MB-231, MCF-7) cell lines. Results showed that LINC00324 was markedly higher in MCF-10A normal breast epithelial cells, which predicts that downregulated expression of LINC00324 shows strong correlation with poor prognosis (Figure 2C). Conversely, no significant correlation was found between LINC00324 expression and age, lymphatic metastasis, ER, PR, Her-2, or Ki-67 (Supplementary Table 2). The Kaplan–Meier Plotter tool analysis (https://kmplot.com) clearly showed that downregulation of LINC00034 was significantly correlated with poor overall survival (Figure 2D).

### Overexpression of LINC00324 attenuates the biological activities of MDA-MB-231 breast cancer cells

**Figure 3:**
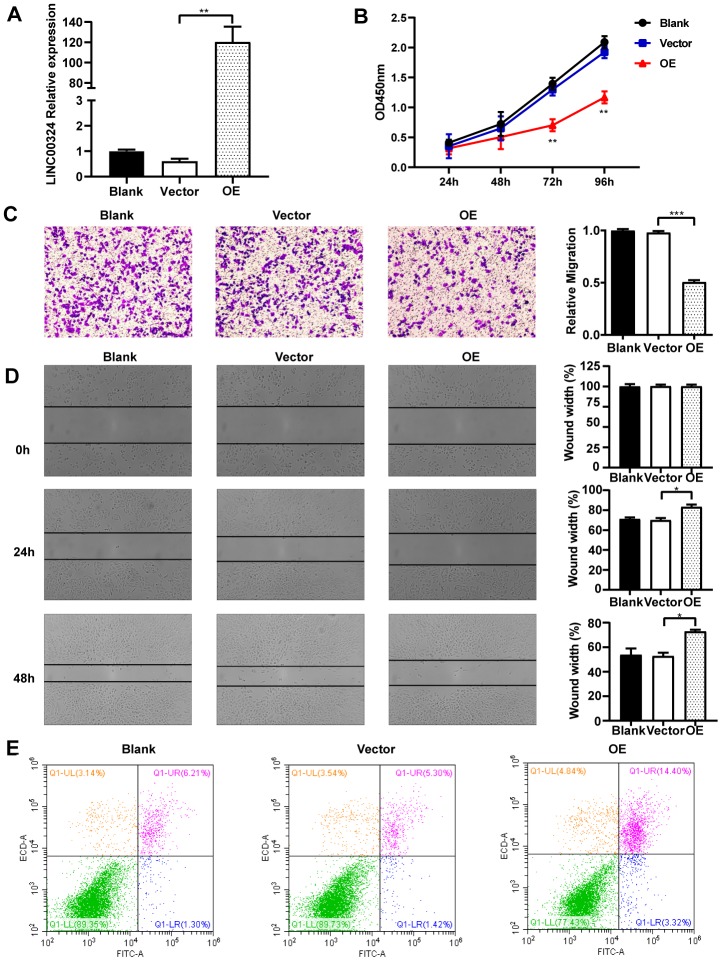
**Overexpression of LINC00324 attenuates the cancer-related biological characteristics of MDA-MB-231 cells.** (**A**) Lentivirus-mediated LINC00324 overexpression vectors were constructed and validated by qRT-PCR. (**B**) Proliferation of MDA-MB-231 cells in response to LINC00324 overexpression, measured using MTT assay. (**C**, **D**) Representative images of transwell assay showing MDA-MB-231 cells’ migration across the membrane after LINC00324 overexpression. Shown are images of immunohistochemical staining and the scratch wound healing assay. The histograms show the average number of migrated cells per field, calculated from five representative fields. (**E**) Apoptosis was assessed by flow cytometry in MDA-MB-231 cells after overexpression of LINC00324. All data are shown as means ± SEM. * *P* < 0.05, ** *P* < 0.01, *** *P* < 0.001. Data are from three independent experiments (**A**, **B**), or are representative of three independent experiments with similar results (**C**–**E**).

*In vitro* experiments were designed to explore the cellular function of LINC00324 *i*n breast cancer cell lines. As shown in Figure 2C, MDA-MB-231 cells had lower expression of LINC00324, when compared to MCF-7 and MCF-10A cells. We further verified the biological effects of LINC00324 overexpression in the MDA-MB-231 cell line. Lentivirus-mediated LINC00324 overexpression vectors were constructed and validated by qRT-PCR and fluorescence evaluation (Figure 3A and Supplementary Figure 1). LINC00324 overexpression showed dramatic inhibition of MDA-MB-231 cells proliferation (Figure 3B). Transwell-matrigel assay demonstrated that invasive ability was impaired following overexpression of LINC00324 in MDA-MB-231 cells (Figure 3C). In subsequent cell migration experiments, the migration capacity was restrained after transfecting the cells with LINC00324 overexpression vectors (Figure 3D). In addition, flow cytometry analysis revealed that enhanced apoptosis occurred in MDA-MB-231 cells transfected with LINC00324 overexpression plasmids, when compared with cells transfected with empty vectors (Figure 3E). These results suggest that LINC00324 might serve as a tumor suppressor by restricting its ability to proliferate, invade, and migrate, as well as inducing apoptosis in MDA-MB-231 cells.

### LINC00324 knockdown promotes cell growth of MCF-7 breast cancer cells

**Figure 4:**
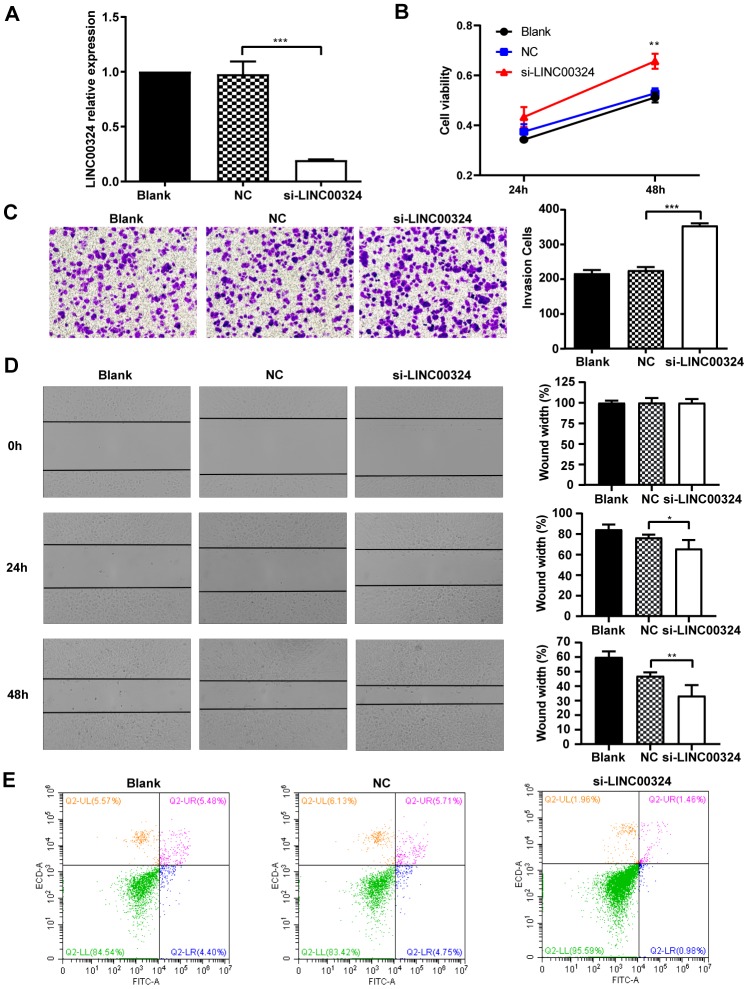
**LINC00324 knockdown promotes the proliferative ability of MCF-7 cells.** (**A**) qRT-PCR assays for LINC00324 levels in MCF-7 cells transfected with siRNA targeting LINC00324. (**B**) MCF-7 cells proliferation was detected by MTT assay after LINC00324 knockdown. (**C**) Transwell assays performed with MCF-7 cells transfected with LINC00324 siRNA or with negative control siRNA. (**D**) Wound healing assay was performed to determine the migration ability of MCF-7 cells after being transfected with LINC00324 siRNA or with negative control siRNA. (**E**) Flow cytometry analysis of the percentage of apoptotic MCF-7 cells with LINC00324 knocked-down. * *P* < 0.05, ** *P* < 0.01, *** *P* < 0.001. All data are shown as means ± SEM. Data are from three independent experiments (**A**, **B**), or are representative of three independent experiments with similar results (**C**–**E**).

To further investigate whether LINC00324 expression is sufficient for tumor suppression, loss-of-function experiments were performed on MCF-7 breast cancer cells. Pre-designed siRNA, targeted to LINC00324 (si-LINC00324), was synthesized and verified by qRT-PCR (Figure 4A). Based on the MTT assay, significantly increased cell viability was observed in MCF-7 cells transfected with si-LINC00324, compared to negative control cells (Figure 4B). The invasion ability of MCF-7 cells was markedly reinforced after expression of LINC00324 had been silenced (Figure 4C). Furthermore, it was noted, when evaluating colony formation, that silencing of LINC00324 expression resulted in enhanced clone generating ability in MCF-7 cells, indicating increased proliferation of these cells (Supplementary Figure 2A). Wound healing assays demonstrated that the migratory potential of LINC00324-silenced cells was significantly increased compared with that of control siRNA-treated MCF-7 cells (Figure 4D). Furthermore, flow cytometric analysis showed apoptosis level to be depressed in MCF-7 cells transfected with si-LINC00324. These results suggest that the observed increased in proliferation was probably due to inhibition of apoptosis in MCF-7 cells (Figure 4E). In addition, knockdown of LINC00324 barely influenced the cell cycle arrest in MCF-7 cells (Supplementary Figure 2B).

### LINC00324 directly interacts with miR-10b-5p to regulate breast cancer progression

**Figure 5:**
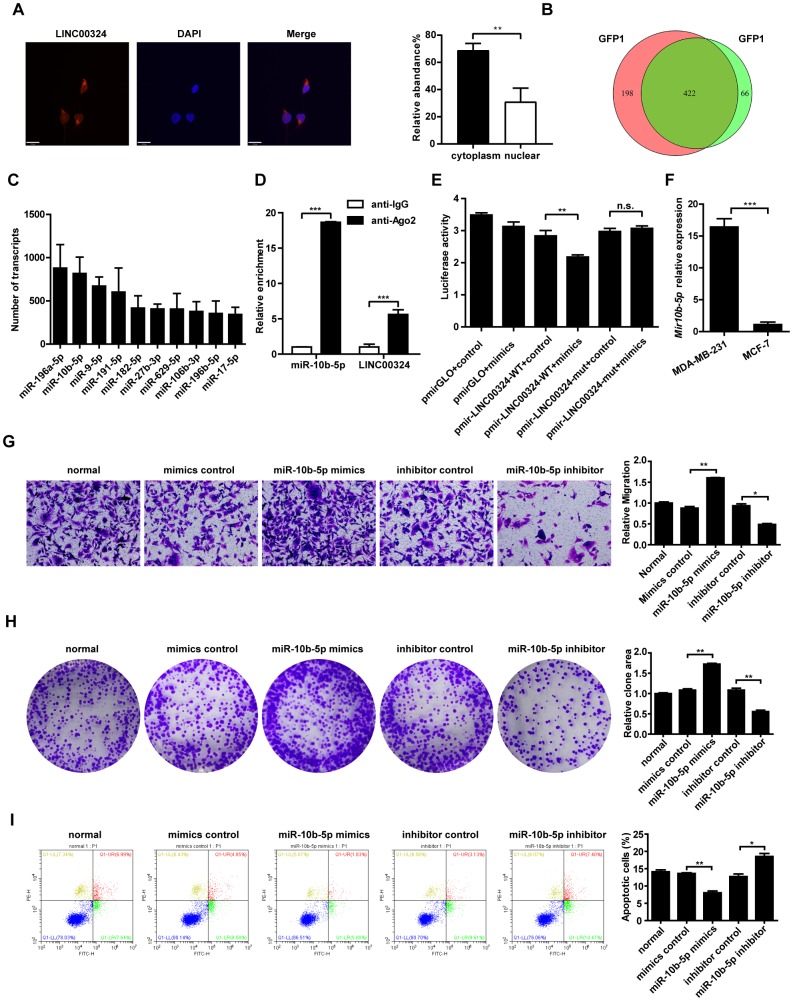
**Candidate miRNAs scanning, verification, and function correlation with LINC00324.** (**A**) FISH assay was performed to detect the subcellular distribution of LINC00324 in MDA-MB-231 cells. The LINC00324 probe mix and control RNA probe mix were labeled with Cy3. DAPI was used to counterstain the nuclei. The high resolution images were captured with a laser scanning confocal microscope. (**B**) Wayne diagram indicates the overlap microRNA transcrips of two independent groups. (**C**) Top ten potential LINC00324 bound reads from MS2-RIP-seq. (**D**) RIP assays showing the association of LINC00324 with miR-10b-5p in MDA-MB-231 cells. (**E**) MDA-MB-231 cells were co-transfected with LINC00324- 3′ UTR wild type or LINC00324- 3′ UTR mutation reporter plasmids, together with miR-10b-5p mimic or mimic negative control and then subjected to the luciferase assay. (**F**) Relative expression of miR-10b-5p in MDA-MB-231 and MCF-7 cells. (**G**) Migration ability of MDA-MB-231 cells determined by Transwell assay. (**H**) Colony formation assays performed with MDA-MB-231 cells transfected with miR-10b-5p mimics, miR-10b-5p inhibitor, or negative control. (**I**) Flow cytometry analysis of the percentage of apoptotic MDA-MB-231 cells with miR-10b-5p mimics or miR-10b-5p inhibitor. All data are shown as means ± SEM. * *P* < 0.05, ** *P* < 0.01, *** *P* < 0.001. Data are from three independent experiments (**D**–**F**), or are representative of three independent experiments with similar results (**A**, **G**, **H**, **I**).

We aimed to screen for potential target genes of LINC00324. Previous studies had demonstrated that lncRNAs may play their role via reciprocal interaction with other regulatory proteins. RNA pull-down assay revealed that LINC00324 showed no association with protein expression of vimentin and Ewing sarcoma (EWS) RNA binding protein 1 (data not shown). As lncRNA could serve as competitive endogenous RNA or sponge for miRNAs, and thus exerting its biological function, we searched for potential miRNA targets of LINC00324 that are involved in promoting breast cancer progression. Fluorescence in situ hybridization was performed, showing that the subcellular distribution of LINC00324 was predominantly in the cytoplasm (Figure 5A). MS2-RIP-seq approach was employed to pull down endogenous mRNAs associated with LINC00324 and the retrieved RNA was sequenced. The Wayne diagram showed 422 overlapped miRNA enriched transcripts in two independent samples (Figure 5B). We further selected the top-ranking potential binding sites of 10 pro-cancer miRNAs enriched transcripts within the LINC00324 sequence for subsequent experiments (Figure 5C). miR-10b-5p has attracted our attention as a potential miRNA target of LINC00324. Subsequently, AGO2-RIP assay, followed by qRT-PCR analysis, demonstrated that miR-10b-5p and LINC00324 were both immunoprecipitated by AGO2, indicating that miR-10b-5p and LINC00324 potentially interacted in an RNA-induced silencing complex (Figure 5D). To further clarify the detailed interaction between miR-10b-5p and LINC00324, a luciferase reporter assay was performed. As shown in Figure 5E, overexpression of miR-10b-5p decreased the luciferase activity of LINC00324 but failed to affect the luciferase activity of 3'-UTR-mutant LINC00324, suggesting that miR-10b-5p interacted with LINC00324 at the predicted binding site. We next evaluated the expression of miR-10b-5p in different breast cancer cell types and results showed a dramatically increased expression in malignant breast cancer cells such as MDA-MB-231 (Figure 5F). We further explored the function of miR-10b-5p in breast cancer cells. The migration ability of MDA-MB-231 cells had dramatically increased in the presence of miR-10b-5p mimics, and decreased after treatment with miR-10b-5p inhibitor (Figure 5G). Colony formation detection showed enhanced clone generation ability of MDA-MB-231 cells after miR-10b-5p mimics delivery. This indicated enhanced proliferation of MDA-MB-231 cells. Moreover, treatment with miR-10b-5p inhibitor resulted in a significant decrease in colony formation of MDA-MB-231 cells (Figure 5H). Flow cytometric analysis indicated that when transfected with miR-10b-5p mimics, apoptosis level of MDA-MB-231 cells had decreased. On the other hand, when treated with miR-10b-5p inhibitor, apoptosis level had markedly increased (Figure 5I). Taken together, results suggest that LINC00324 directly interacts with miR-10b-5p. This interaction results in interference in miR-10b-5p’s function in a competitive binding pattern.

### LINC00324 upregulates the expression of E-cadherin by sponging miR-10b-5p

**Figure 6:**
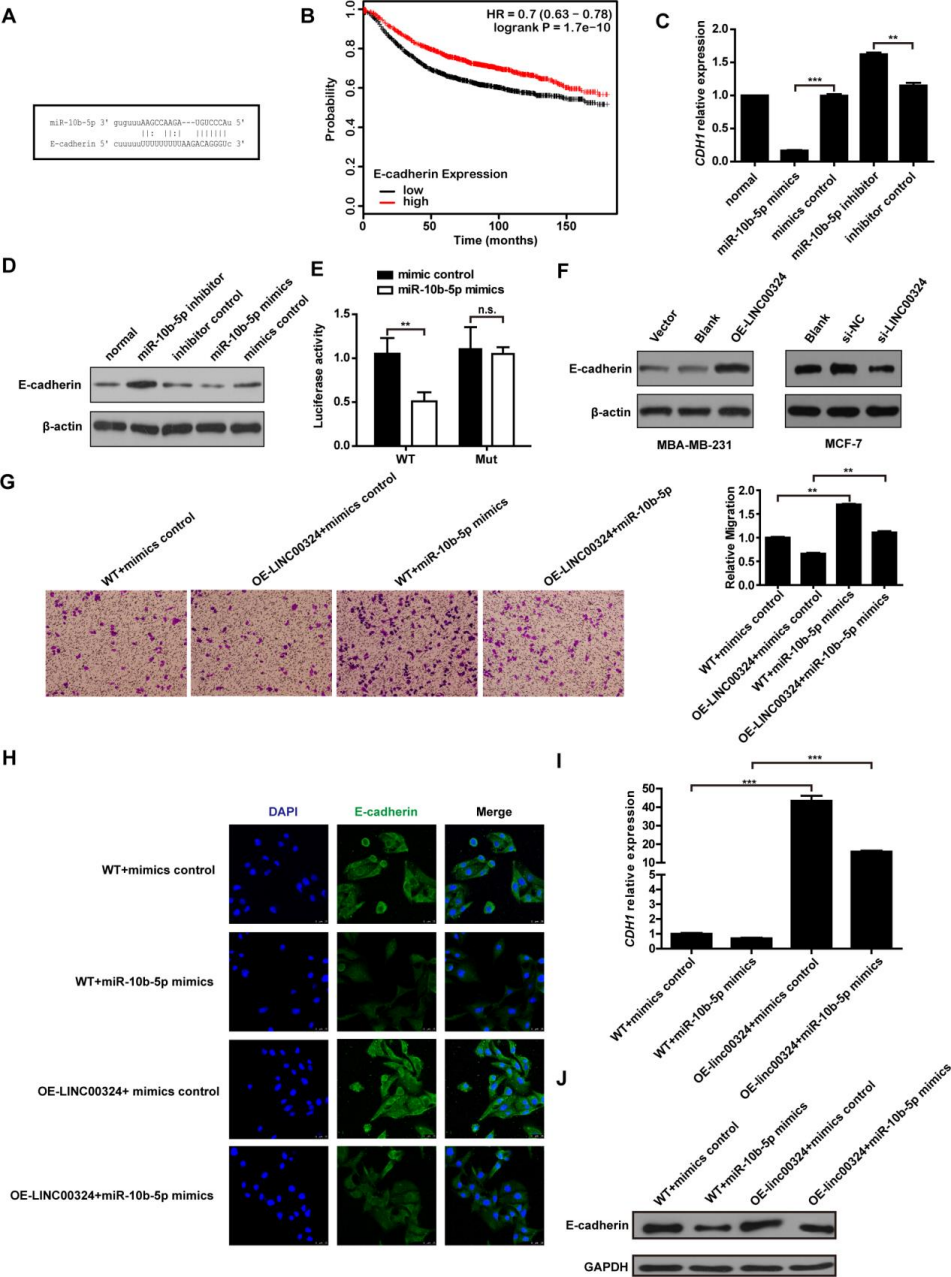
**LINC00324 restored E-cadherin expression by sponging miR-10b-5p.** (**A**) Binding site of miR-10b-5p on E-cadherin. (**B**) Kaplan–Meier analysis for overall survival based on low and high E-cadherin levels (the KM Plotter database). (**C**) Relative expression of *CDH1* in MDA-MB-231 cells transfected with miR-10b-5p mimics or inhibitor. (**D**) Western blot analysis of E-cadherin protein expression in MDA-MB-231 cells after transfection with miR-10b-5p mimics or inhibitor. (**E**) Luciferase assay of miR-10b-5p overexpression MDA-MB-231 cells transfected with pmirGLO-3′ UTR reporter of E-cadherin. (**F**) Western blot analysis of E-cadherin protein expression in MDA-MB-231 or MCF-7 cells after transfection with LINC00324 overexpression vectors or LINC00342 siRNA. (**G**) Transwell assay indicating migration of MDA-MB-231 cells after transfection with LINC00324 overexpression vectors and miR-10b-5p mimics. (**H**) Immunofluorescence staining of E-cadherin (Green) in indicated MCF-7 cells. Scale bar = 50 μm. (**I**, **J**) qRT-PCR analysis of *CDH1* mRNA level (**I**) or western blot analysis of E-cadherin protein level (**J**) in indicated MCF-7 cells. All data are shown as means ± SEM. * *P* < 0.05, ** *P* < 0.01, *** *P* < 0.001. Data are from three independent experiments (**C**, **I**), or are representative of three independent experiments with similar results (**D**, **F**–**H**, **J**).

To further investigate the possible associated mechanism involved in breast cancer progression, the MiRanda database (http://www.microrna.org/microrna/home.do) was consulted, aiming to predict the potential target gene of miR-10b-5p. The bioinformatics database suggested that E-cadherin showed a potential binding site for miR-10b-5p (Figure 6A). The Kaplan–Meier Plotter tool analysis indicated that high level of E-cadherin represents a positive prognostic probability for breast cancer patients (Figure 6B). We further evaluated whether miR-10b-5p affects the expression of E-cadherin in MDA-MB-231 cells. Results showed that both protein and mRNA levels of E-cadherin had decreased when miR-10b-5p was overexpressed, and had increased when miR-10b-5p was inhibited in MDA-MB-231 cells (Figure 6C and 6D). A further dual-luciferase reporter assay indicated that miR-10b-5p directly binds to E-cadherin to regulate its expression (Figure 6E). We then looked into whether LINC00324 regulated the expression of E-cadherin in MBA-MD-231 cells in a miR-10b-5p-dependent manner. Western blot analysis showed a significant increase in the expression of E-cadherin when LINC00324 was overexpressed in MDA-MB-231 cells (Figure 6F). In addition, western blot analysis indicated that the expression of E-cadherin was significantly reduced after transfection with si-LINC00324 in MCF-7 cells (Figure 6F). Transwell assay results indicated that migration induction by miR-10b-5p was significantly reversed when LINC00324 was overexpressed in the MDA-MB-231 cells (Figure 6G). Besides, immunofluorescence (IF) staining indicated that expression of E-cadherin decreased when miR-10b-5p mimics were overexpressed. This downregulation of E-cadherin mRNA and protein expression levels was significantly reversed by LINC00324 overexpression in MCF-7 cells (Figure 6h-6J). In addition, cells morphology tended toward epithelial phenotype after LINC00324 overexpression (Supplementary Figure 3). These results indicate that LINC00324 regulates E-cadherin expression through direct targeting of miR-10b-5p.

### LINC00324 inhibits tumorigenicity of breast cancer cells

**Figure 7:**
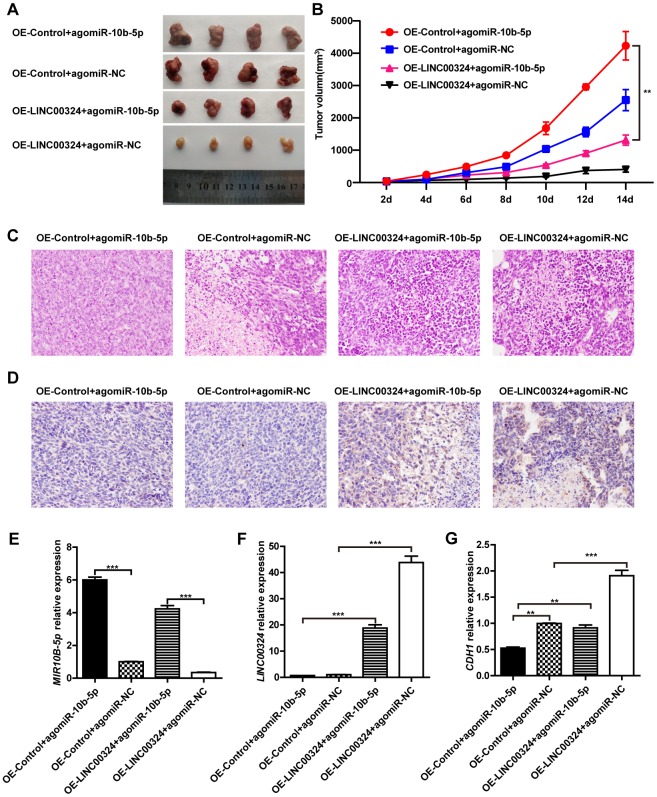
**LINC00324 inhibited tumor growth in nude mice xenograft models.** (**A**) Dissection of tumors from non-obese diabetic (NOD) mice xenografted with agomiR-10b-5p alone or combined with overexpressed-LINC00324 MDA-MB-231 cells. (**B**) Tumor volume curve of overexpressed-LINC00324 and agomiR-10b-5p treatment groups were measured and analyzed. (**C**) Hematoxylin and eosin-stained images of breast cancer tissues from NOD mice xenografted with agomiR-10b-5p alone or combined with overexpressed-LINC00324 MDA-MB-231 cells. (**D**) Immunohistochemical staining of E-cadherin expression in breast cancer tissues from NOD mice xenografted with agomiR-10b-5p alone or combined with overexpressed-LINC00324 MDA-MB-231 cells. (**E**–**G**) Relative expression of miR-10b-5p, LINC00324, and CDH1 in breast cancer tissues from NOD mice xenografted with agomiR-10b-5p alone or combined with overexpressed-LINC00324 MDA-MB-231 cells. All data are shown as means ± SEM. ** *P* < 0.01, *** *P* < 0.001. Data are from three independent experiments (**B**, **E**–**G**), or are representative of three independent experiments with similar results (**A**, **C**, **D**).

We further validated the function of LINC00324 *in vivo*. LINC00324-overexpressing MDA-MB-231 cells were subcutaneously transplanted into nude mice, while miR-10b-5p agomir was delivered into nude mice through tail vein injection. After tumor xenograft, the tumor weight and volume were recorded at the indicated time points, and tumor tissues were collected after the last recording. Results showed that tumor weight and volume were significantly reduced in the LINC00324 overexpression group, when compared with control (Figure 7A and 7B). Consistent with this finding, hematoxylin and eosin staining showed that tumorigenicity was dramatically attenuated in the LINC00324 overexpression group (Figure 7C). Meanwhile, immunohistochemical (IHC) staining showed that expression of E-cadherin had markedly increased in the LINC00324 overexpression group (Figure 7D). We further verified the expression of miR-10b-5p and LINC00324 in breast cancer cells *in vivo*. As shown in Figure 7E and 7F, the mRNA level of miR-10b-5p was significantly higher in the agomiR-10b-5p group, and the expression of LINC00324 was higher in the LINC00324 overexpression group. Moreover, expression of E-cadherin had significantly decreased after miR-10b-5p was delivered, and this was reversed after overexpression of LINC00324 (Figure 7G). These data demonstrate that LINC00324 overexpression restricts the progression of breast cancer and may serve as a potent tumor suppressor in breast cancer treatment.

## DISCUSSION

With the high occurrence and metastasis rate, breast cancer has become a major factor threatening women's health [16]. Previous studies have demonstrated that lncRNAs play a critical role in physiological and pathological processes of various malignant cancers, including breast cancer [17]. The functions of LINC00324, one such lncRNA, in breast cancer remains unknown. In this study, decreased expression of LINC00324 was shown to occur in breast cancer sequences retrieved from the TCGA and GEO databases. Consistent with the bioinformatics analysis results, down-regulation of LINC00324 was observed in breast cancer tissues and malignant breast cancer cells. Aberrant expression of LINC00324 is associated with malignant clinicopathological characteristics of the disease and suggests a possible regulatory capacity in breast cancer.

Emerging evidence indicates that most lncRNAs play pro-cancer roles in breast cancer progression, with diverse underlying mechanisms. Only few anti-cancer lncRNAs have been identified in breast cancer [18]. Here, we have demonstrated that LINC00324 serves as a suppressor in breast cancer development and progression. The following evidence support our conclusion. To begin with, overexpression of LINC00324 exhibited inhibition ability on the proliferation, invasion, and migration of malignant breast cancer cells, while apoptosis level had significantly increased. Furthermore, loss-of-function approach indicated that decreased expression of LINC00324 contributed to enhanced biological activity of benign breast cancer cells. Consistently, overexpression of LINC00324 showed a significant inhibitory effect on breast cancer tumor growth *in vivo*. These findings suggest that LINC00324 is capable of restraining breast cancer progression in malignant breast cancer cells, while its downregulation leads to aberrant activation of cancer-related biological processes. It can thus be suggested that LINC00324 is an important anti-cancer lncRNA, involved in the suppression of breast cancer development and progression, both *in vitro* and *in vivo*. Recent studies have suggested that LINC00324 plays a pivotal role in modulating osteosarcoma and gastric cancer progression [19, 20]. Although these findings suggest that LINC00324 suppresses the development and progression of diverse cancers that already exist, there is no proof to associate LINC00324 with the initial onset of cancer. Ours and others results illustrate that LINC00324 may have different regulatory pattern and function in different types of cancer. With the development of nanotechnology and gene therapy, targeting specific diseased tissue or organ is becoming a reality. Exogenous LINC00324 targeted delivery for clinical breast cancer therapy is now a viable possibility.

LncRNAs regulate gene expression at different levels, including chromatin modification, and transcriptional and post-transcriptional regulation [21]. In subsequent mechanism exploration, fluorescence in situ hybridization revealed that LINC00324 is mainly localized to the cytoplasm, suggesting it exerts there a post-transcriptional regulatory function. Our results also indicate that LINC00324 is barely influenced by cell cycle, suggesting that it is not associated with cell cycle-related mRNAs and proteins. Furthermore, our current study shows that LINC00324 failed to bind with downstream regulatory proteins like vimentin and EWS RNA binding protein 1. A mechanism pattern in which lncRNA serves as decoy miRNA binding site, like competing endogenous RNA (ceRNA) or sponge, to control the functions of regulatory miRNA is attracting great attention [22]. In line with this, we sought for miRNAs that would potentially bind LINC00324. Following RIP-seq and RIP-PCR assays, miR-10b-5p drew our attention as a target candidate miRNA. miR-10b-5p is a highly conserved short non-coding RNA strand that is involved in post-transcriptional gene silencing in multiple biological processes. It affects the stability and translation of mRNAs. Previous studies have demonstrated that high expression of miR-10b-5p contributes to breast cancer progression and metastasis [23, 24]. For instance, the exosome-mediated transfer of miR-10b from breast cancer to adjacent and distant normal cells can induce tumor development and progression in those cells [25]. Moreover, restricting the expression of miR-10b, with a kind of antagomir, appears to prevent metastasis formation [26]. It is well known that cancer growth depends on intratumoral angiogenesis. Overexpression of miR-10b can promote angiogenic behavior in cultured human endothelial cells [27], which may be one explanation why miR-10b promotes cancer growth. Interestingly, polymer nanoparticles-mediated targeting of miR-10b, causing its down-expression, exhibited substantial reduction in triple negative breast cancer growth [28]. Consistent with these reports, luciferase assay confirmed that LINC00324 can directly bind to miR-10b-5p. We have also demonstrated here that antagonizing miR-10b-5p with LINC00324, attenuates biological processes associated with breast cancer, suggesting potential usefulness of this route as a clinical therapy strategy.

Polarized epithelial cells complete multifaceted changes that cause them to begin expressing a mesenchymal phenotype. This process is referred to as the epithelial–mesenchymal transition (EMT) [29]. Cancer progression and metastasis rely on a series of changes, including the EMT process, which increases cell motility, invasiveness, and results in the dissemination of primary tumor cells to distant anatomic sites [30]. Mechanistically, our further exploration indicates that E-cadherin is a downstream molecular target of miR-10b-5p, and results showed a direct interaction. E-cadherin, a calcium-dependent cell-cell adhesion protein, plays a pivotal role in maintaining intercellular tight junctions [31]. Loss of E-cadherin expression results in an acquisition of a more differentiated epithelial phenotype and participation in EMT transformation of cancer [32]. Cancer cells with EMT phenotype show attenuated intercellular junction, increased proliferation and invasion, and high frequency of distant metastasis [33]. Our results show that overexpression of miR-10b-5p exerts a restrain on E-cadherin levels, while this inhibitory effect is partially removed by LINC00324. Therefore, maintaining E-cadherin is presently considered to be a promising strategy to inhibit aggressive cancer phenotypes, invasion, and metastasis, and improve patients’ survival.

Taken together, we found that LINC00324 is significantly downregulated in malignant breast cancer and is associated with the clinical and pathological characteristics of the disease. LINC00324 promotes E-cadherin expression by directly targeting miR-10b-5p via a sponge pattern, and subsequently restraining the progression of breast cancer. Finally, our results indicate that LINC00324 can be a potent candidate marker for clinical diagnosis and can be used as a therapeutic agent for breast cancer.

## MATERIALS AND METHODS

### Cell culture and clinical tissue samples

All cell lines involved in the *in vivo* and *in vitro* experiments were obtained from the American Type Culture Collection (ATCC, Manassas, VA, USA). Cell lines were cultured in Dulbecco’s modified Eagle’s medium (DMEM) (Hyclone, Utah, USA) with 4.5 g/L of glucose and supplemented with 10% fetal bovine serum (FBS; Gibco, CA, USA). A total of 45 breast cancer tissue samples, and paired adjacent normal tissue samples, were obtained from the First Affiliated Hospital of Guangxi Medical University. Collection of all human samples used in the experiments was approved by the Medical Ethics Committee of Guangxi Medical University. Informed consent for participation in this study was obtained from all patients.

### RNA isolation and quantitative RT-PCR

Total RNA was extracted from cells and tissues using TRIzol reagent (Invitrogen, CA, USA) according to the manufacturer’s instructions. Purified RNA samples were reverse-transcribed into cDNA with RevertAid First Strand cDNA Synthesis Kit (Thermo Fisher Scientific, Waltham, MA, USA). qRT-PCR was performed using 7900HT Fast Real-Time PCR System (Applied Biosystems, Thermo Fisher Scientific, Waltham, MA, USA) using SYBR^®^ Premix Ex Taq^TM^ II (Takara, Dalian, China). Data were normalized to β-actin (*ACTB*) expression. The primers used were as follows: *LINC00324*: forward: 5′-AGTCCTGAAGGCACTCTGTCTC-3′ and reverse: 5′-ATCCCAACCAACCACTATACTAACG-3′; *ACTB*: forward: 5′- CATGTACGTTGCTATCCAGGC-3′ and reverse: 5′- CTCCTTAATGTCACGCACGAT-3′; *CDH1*: forward: 5′- GCCCTGCCAATCCCGATG-3′ and reverse: 5′- CAGACTAGCAGCTTCGGAAC-3′

Relative fold expressions were calculated using the comparative threshold cycle (2^-ΔΔCt^) method.

### Western blot

Total protein of cultured cells was isolated using RIPA lysis buffer (Beyotime, Shanghai, China). Concentration of proteins was measured with BCA Protein Assay Reagent Kit (Thermo Fisher Scientific, Waltham, MA, USA). Equal amounts of protein samples were separated by SDS-PAGE and transferred onto polyvinylidene difluoride (PVDF) membrane. The membrane was incubated with 5% non-fat milk in TBST for 1 h at room temperature. Samples were then incubated with anti-E-cadherin (Abcam, ab194982) and anti-β-actin (Abcam, ab8226) primary antibodies at 4 °C overnight. Images were acquired after 1 h incubation with horseradish peroxidase-conjugated secondary antibody at room temperature.

### Transwell invasion assays

Transwell invasion assay was performed to investigate cells invasion ability. The assay was done in 24-well Transwell plates (Corning) with 8 μm-pore inserts coated with Matrigel (Corning). MCF-7 and MDA-MB-231 cells in serum-free DMEM were seeded into the upper chamber, while DMEM supplemented with 10% FBS was added to the lower chamber. After 24 h incubation, the Matrigel and the cells remaining in the upper chamber were removed with a cotton-tipped swab. Then, the cells in the lower chamber were fixed with 4% paraformaldehyde for 15 min, stained with crystal violet for 20 min, and then washed with PBS. The number of invasive cells in five random fields (×200) was counted under a light microscope (Leica).

### Wound healing assay

The migratory ability of MCF-7 and MDA-MB-231 cells was detected by the scratch wound healing assay. Cells were seeded into 6-well plates (~5×103 cells/well) for 24 h. The monolayers were scratched using a 200 μL pipette tip and then the floating cells were removed by washing with PBS. Subsequently, the cells were incubated in serum-free DMEM for 24 h, 48 h, and 72 h, and the cells’ migration was monitored with a camera connected to a microscope. The percentage of wound healing was observed under an optical microscope and analyzed using ImageJ software (Bethesda, USA).

### Cell cycle and apoptosis analysis

Flow cytometric analysis was performed to study cell cycle distribution and apoptosis level. Briefly, to carry out the cell cycle and apoptosis assays, treated cells were harvested and then washed twice with pre-cooled PBS. For cell cycle analysis, cells were fixed with 75% ice-cold ethanol at 4 °C overnight, followed by incubation with propidium iodide and RNase A for 15 mins at 37 °C. After washing with cold PBS three times, FACS caliber flow cytometry (Beckman Cytoflex) was used to acquire the DNA contents of labeled cells. For apoptosis analysis, Annexin V-fluorescein isothiocyanate (FITC)/PI straining was performed and sample acquisition was done using flow cytometry according to the manufacturer’s instructions (Beyotime, Shanghai, China). Collected data was analyzed with Flow Jo software.

### Colony formation analysis

After being incubated for 48 h, the transfected single-cell suspensions were seeded into six-well plates (200 cells/well) and incubated in an incubator with 5% CO_2_ at 37 °C. After 2 weeks of incubation, the cells were fixed with 4% paraformaldehyde and stained with 0.1% crystal violet. The number of the colonies was counted by optical microscope.

### Cell proliferation assay

MTT (Beyotime, Shanghai, China) colorimetric assay was performed to assess cells proliferation. Briefly, cells in the logarithmic phase were collected, and their concentration was adjusted. The cells were seeded in a 96-well plates at 5×10^5^ cells per well, and cultured for 24 h,48 h,72 h, and 96 h at 37 °C in humidified incubator with atmosphere of 5% CO_2_ in air. MTT solution (10 μL) was added to the medium, and the cells were incubated for another 4 h at 37 °C. Following that, 100 μL Formazan solution were added and plates were incubated for another 3-4 h at 37°C. The cells proliferation curves were plotted using the absorbance at each time point.

### RNA immunoprecipitation (RIP) sequencing assay

MDA-MB-231 cells were co-transfected with pcDNA3.1-LINC00324 and pMS2-GFP (Addgene plasmids). After 48 h, the cells were harvested and used for RIP experiments, utilizing an anti-GFP antibody (Abcam, CA, USA) and the Magna RIP™ RNA-Binding Protein Immunoprecipitation Kit (Millipore, Darmstadt, Germany) according to the manufacturer’s instructions. Briefly, cells were collected and lysed in complete radioimmunoprecipitation assay (RIPA) buffer, containing protease inhibitor cocktail and RNase inhibitor. Next, the cells lysates were incubated with RIP buffer, containing magnetic beads conjugated antibody or control normal IgG. The samples were digested with proteinase K to isolate the immunoprecipitated RNA.

The products were purified and enriched to perform Illumina sequencing, seeking potential target microRNA. For anti-AGO2 RIP, MDA-MB-231 cells were used to perform RIP experiments, utilizing an AGO2 antibody (Millipore) as described above, and the purified RNA was finally subjected to real-time PCR to verify the presence of the binding targets [34].

### Plasmid and lentiviral construction

For the lentivirus-mediated overexpression of LINC00324, human full-length DNA of LINC00324 was subcloned into pCDH-CMV-MCS-EF1-GFP lentiviral expression vector. The lentiviral particles were obtained by transfecting 293T cells. The viral supernatant was collected 48h after transfection. For cells transfection, MCF-7 cells were seeded in six-well plates and infected with lentiviral particles expressing LINC00324 overexpression vector and empty vector. The efficiency of infection was determined by fluorescence microscopy and qRT-PCR assays.

### Luciferase reporter assay

Wild-type (WT) or mutant (MUT) LINC00324 binding sites were subcloned into the pmirGLO vector to construct luciferase reporter vector. The miR-10b-5p mimics or control were co-transfected with the constructed pmirGLO or pmir-LINC00324 luciferase reporter vector into HEK-293T cells using Lipofectamine 2000. Luciferase activity was assayed using a Dual Luciferase Reporter Gene Assay kit (Beyotime, Shanghai, China) after cell transfection for 48 h.

### Fluorescence in situ hybridization (FISH)

FISH assay was performed to detect the subcellular localization of LINC00324. RNA FISH was performed using fluorescence in situ hybridization kit (RiboBio, Guangzhou, China) with pre-designed LINC00324 probes labeled with Cy3 fluorescence dye, following the manufacturer’s instructions. Fluorescence detection was performed with a confocal laser-scanning microscope (Leica, Germany).

### Mouse xenografts and immunohistochemistry

The xenograft mice *in vivo* assays were done according to the institutional guidelines and approved by the Animal Ethics Committee of Guangxi Medical University. Nude mice were injected subcutaneously with 0.2 mL of a cell suspension containing 5 × 10^5^ cells. Tumor growth rates were monitored. When a tumor was palpable, it was measured every other day, and the volume was calculated according to the following formula: volume = length × width^2^ × 0.5. For immunohistochemistry staining, tumors were excised and fixed by 4% paraformaldehyde. Serial sections at 6 μm thickness were prepared for immunohistochemistry analysis. Sections were incubated with primary anti-E-cadherin antibody overnight at 4 °C. Corresponding secondary antibodies were used and then stained sections were examined by confocal laser microscopy.

### Statistical analysis

All statistical analyses were performed using GraphPad Prism 7. Student’s t-test was performed for comparison between two groups. For comparison between more than two groups, one-way analysis of variance (ANOVA) with Fisher’s LSD-t test were used. The survival curves were calculated using the Kaplan–Meier method and statistically compared using a log-rank test. *P* < 0.05 was considered statistically significant.

## Supplementary Material

Supplementary Figures

Supplementary Tables
