# Correction for: Long intergenic non-protein coding RNA 324 prevents breast cancer progression by modulating miR-10b-5p

**DOI:** 10.18632/aging.204784

**Published:** 2023-05-31

**Authors:** Bo Wang, Yangyang Zhang, Haitian Zhang, Faquan Lin, Qixin Tan, Qinghong Qin, Wei Bao, Yi Liu, Jiaying Xie, Qiyan Zeng

**Affiliations:** 1 Department of Biochemistry and Molecular Biology, Guangxi Medical University, Nanning 530021, Guangxi, P.R. China; 2 Department of Gastrointestinal and Gland Surgery, The First Affiliated Hospital of Guangxi Medical University, Nanning 530021, Guangxi, P.R.China; 3 Key Laboratory of Biological Molecular Medicine Research, Guangxi Medical University, Nanning 530021, Guangxi, P.R. China; 4 Department of Breast Surgery, Guangxi Medical University Tumor Hospital, Nanning 530021, Guangxi, P.R. China

**Keywords:** breast cancer, LINC00324, miR-10b-5p, E-cadherin, ceRNA

**This article has been corrected:** The authors found that in **Figure 1A** the X-axis labels for the Normal and Cancer groups were wrongly assigned; that labeling has now been corrected. In **Figure 4C**, the transwell assay image of MCF-7 cells transfected with negative control siRNA (NC) was incorrect and was replaced with the correct image from the original set of experiments. These corrections do not change the content of the publication and do not affect the conclusions drawn from this research.

Corrected **Figures 1** and **4** are presented below.

**Figure 1:**
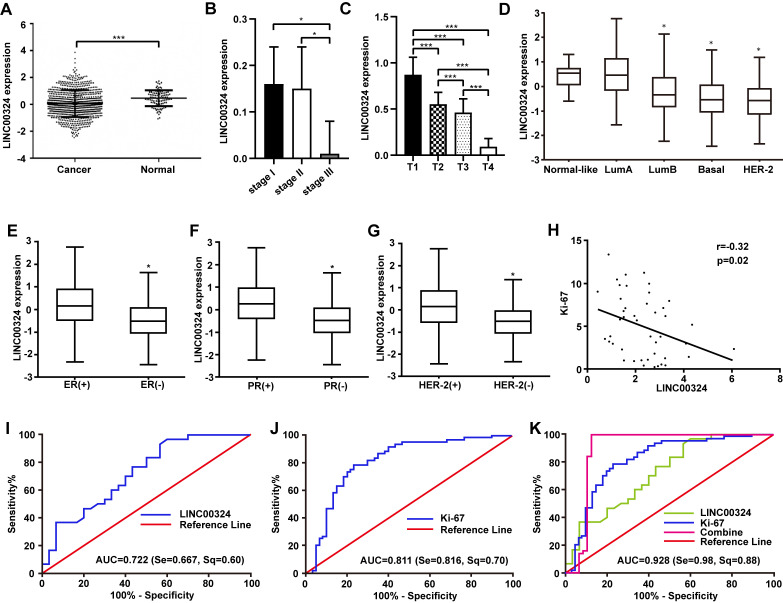
**Highly expressed LINC00324 that is involved in breast cancer was identified by bioinformatics prediction. **(**A**) Relative expression of LINC00324 in normal and breast cancer tissue. (**B, C**) Relative expression of LINC00324 in different clinical stages of breast cancer. (**D**) Relative expression of LINC00324 in five molecular subtypes of breast cancer. (**E**) Relative expression of LINC00324 in estrogen receptor (ER)-positive and -negative breast cancer. (**F**) LINC00324 expression in progesterone receptor (PR)-positive and -negative breast cancer. (**G**) LINC00324 expression in HER-2-positive and -negative breast cancer. (**H**) Pearson’s correlation curve showing the negative correlation between the expression of LINC00324 and Ki-67 in breast cancer. (**I**–**K**) ROC curve for breast cancer diagnostic value of LINC00324 and Ki-6. All data are shown as means ± SEM. * *P* < 0.05, *** *P* < 0.001.

**Figure 4:**
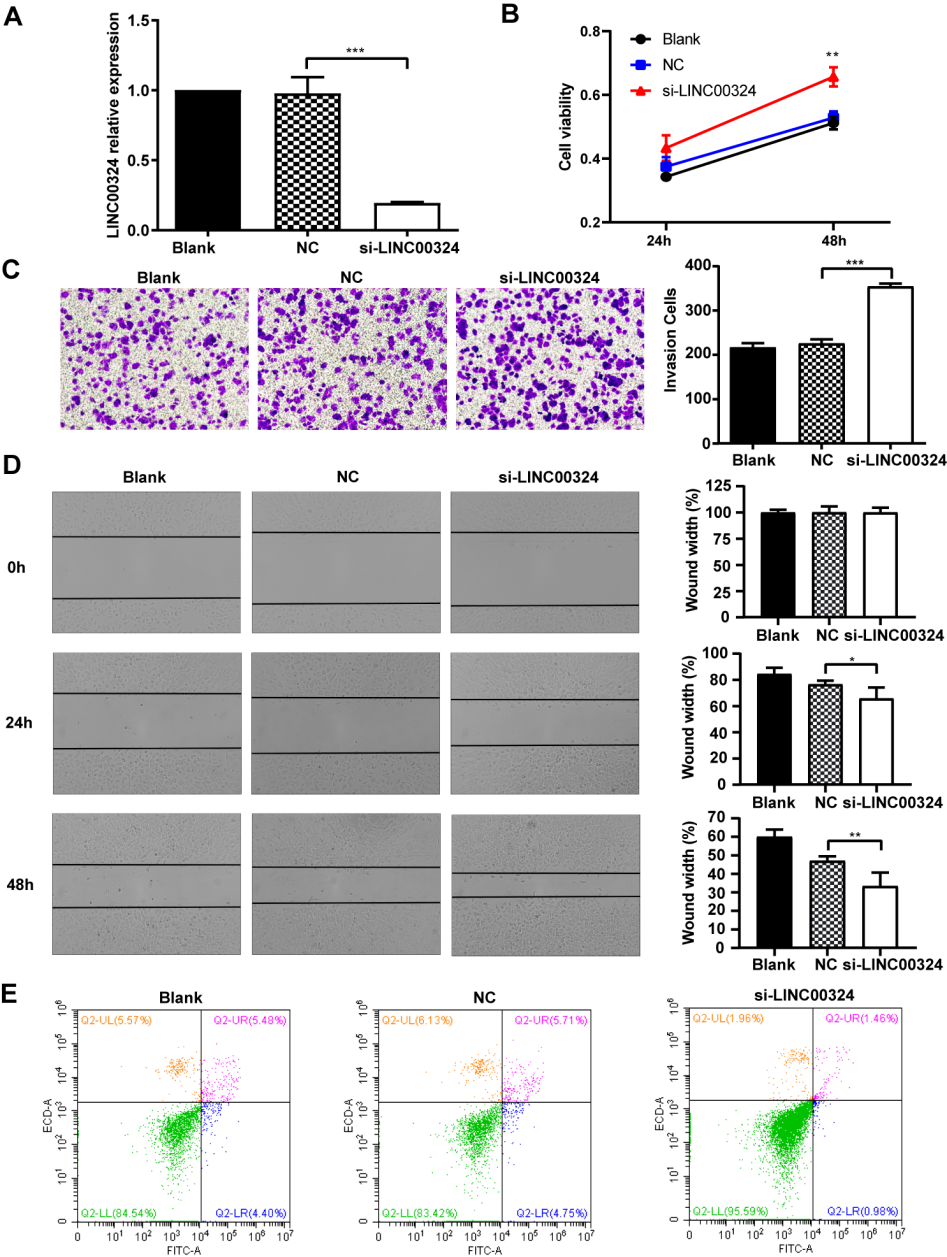
**LINC00324 knockdown promotes the proliferative ability of MCF-7 cells.** (**A**) qRT-PCR assays for LINC00324 levels in MCF-7 cells transfected with siRNA targeting LINC00324. (**B**) MCF-7 cells proliferation was detected by MTT assay after LINC00324 knockdown. (**C**) Transwell assays performed with MCF-7 cells transfected with LINC00324 siRNA or with negative control siRNA. (**D**) Wound healing assay was performed to determine the migration ability of MCF-7 cells after being transfected with LINC00324 siRNA or with negative control siRNA. (**E**) Flow cytometry analysis of the percentage of apoptotic MCF-7 cells with LINC00324 knocked-down. * *P* < 0.05, ** *P* < 0.01, *** *P* < 0.001. All data are shown as means ± SEM. Data are from three independent experiments (**A**, **B**), or are representative of three independent experiments with similar results (**C**–**E**).

