# Supplementary material for: Long intergenic non-protein coding RNA 324 prevents breast cancer progression by modulating miR-10b-5p
Source: Aging (Albany NY). 2020 Apr 18;12(8):6680–99. doi: 10.18632/aging.103021 (PMC7202516; doi:10.18632/aging.103021)
Supplement: Supplementary Figures [file aging-12-103021-s002..pdf]

SUPPLEMENTARY FIGURES

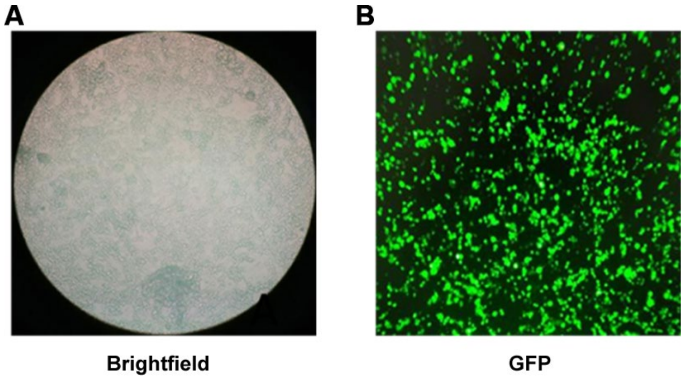

Supplementary Figure 1. The transfection efficiency of LINC00324 in MDA-MB-231 cells. (**A**, **B**) Brightfield and fluorescent microscopy of MDA-MB-231cells stably expressing GFP 48 hours after transfection of LINC00324 overexpression vectors. Data are representative of three independent experiments with similar results.

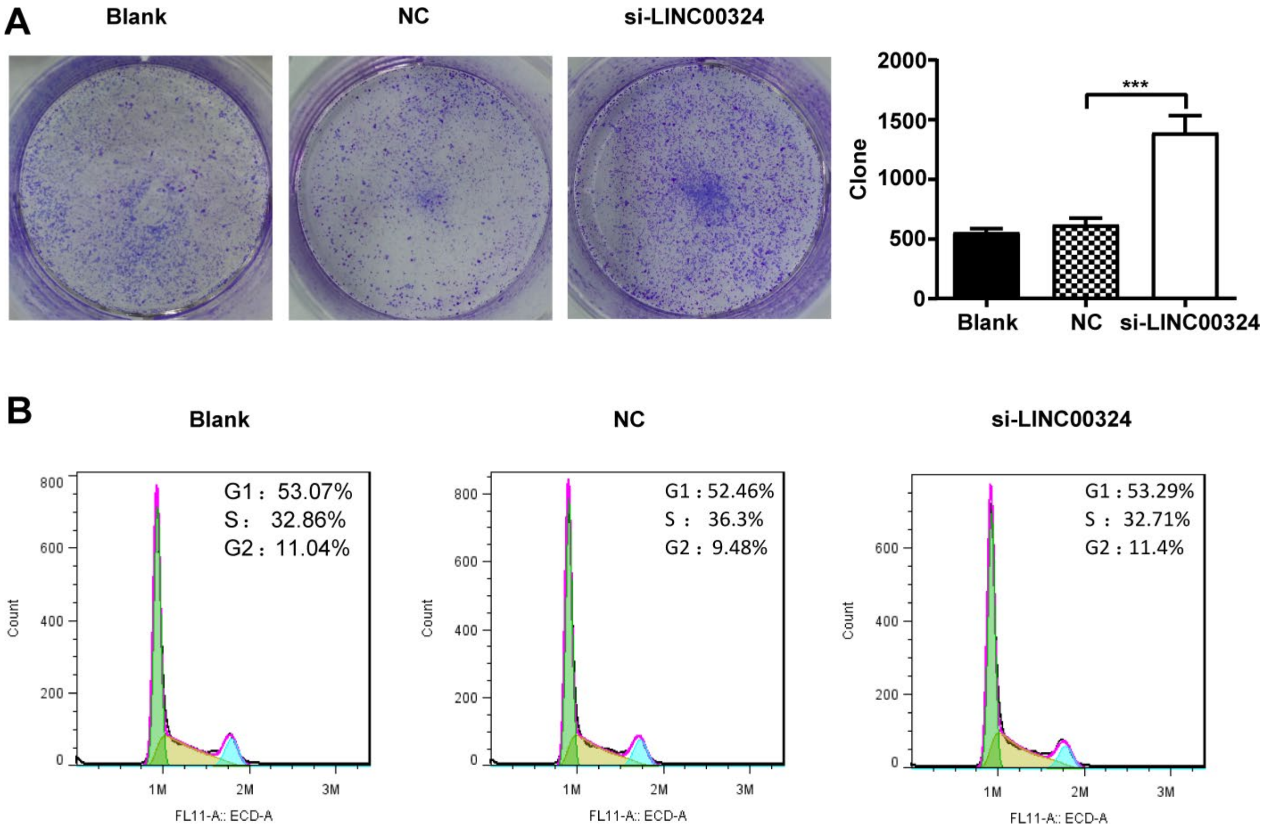

Supplementary Figure 2. Relationship between expression of LINC00324 and proliferation of MCF-7 cells. (**A**) Colony formation assays performed with the MCF-7 cells transfected with LINC00324 siRNA or the negative control siRNA. (**B**) Flow cytometry analysis of the percentage of cell cycle in MCF-7 cells transfected with LINC00324 siRNA or the negative control siRNA. ***$p < 0.001$. Data are from three independent experiments, or are representative of three independent experiments with similar results.

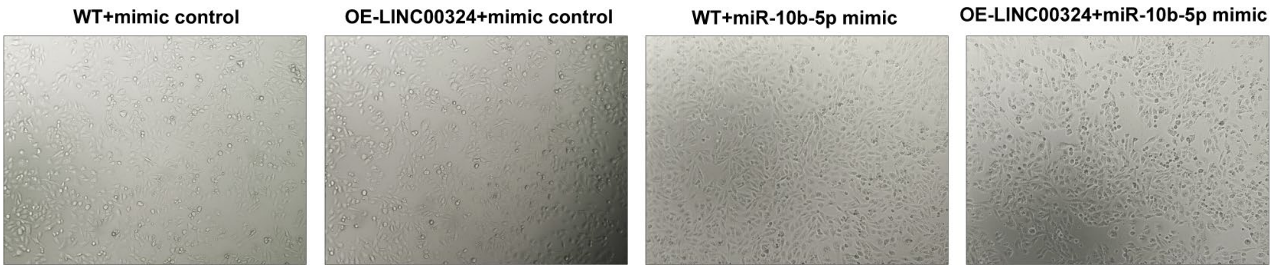

Supplementary Figure 3. MDA-MB-231 cells tends to epithelial phenotype after LINC00324 overexpression. Brightfield microscopy of MDA-MB-231cells in indicated cells. Data are representative of three independent experiments with similar results.
