# Supplementary material for: Long intergenic non-protein coding RNA 324 prevents breast cancer progression by modulating miR-10b-5p
Source: Aging (Albany NY). 2020 Apr 18;12(8):6680–99. doi: 10.18632/aging.103021 (PMC7202516; doi:10.18632/aging.103021)
Supplement: Supplementary Tables [file aging-12-103021-s001..pdf]

SUPPLEMENTARY TABLES

Supplementary Table 1. Correction between LINC00324 expression and clinicopathologic characteristics of breast cancer from TCGA and GEO database.

Characteristics	n	LINC00324 level	P value
TNM Stage			0.019
I	183	0.16±0.08	
II	528	0.15±0.09	
III	249	0.01±0.07	
Primary Tumor (T)			<0.0001
T_1	58	0.87±0.19	
T_2	724	0.55±0.13	
T_3	128	0.46±0.15	
T_4	40	0.09±0.09	
Regional Lymph Nodes (N)			0.155
N_0	399	0.17±0.12	
N_1	364	0.17±0.14	
N_2	120	0.14±0.12	
N_3	77	0.13±0.11	
Distant Metastasis (M)			0.082
M_0	775	0.16±0.08	
M_1	22	0.07±0.21	
M_X	163	0.05±0.22	

Supplementary Table 2. Correction between LINC00324 expression and clinicopathologic characteristics of breast cancer patients.

Characteristics	n	LINC00324 level	P value
Age(years)			0.366
>35	24	0.50±0.51	
≤35	15	0.65±0.40	
TNM Stage			<0.0001
T_1	8	0.87±0.49	
T_2	18	0.66±0.06	
T_3	6	0.28±0.08	
T_4	7	0.09±0.06	
Lymph node metastasis			0.811
Yes	27	0.59±0.61	
No	12	0.53±0.28	
ER			0.866
positive	30	0.59±0.51	
negative	9	0.56±0.47	
PR			0.881
positive	22	0.55±0.38	
negative	17	0.58±0.68	
Her-2			0.266
positive	34	0.64±0.56	
negative	5	0.26±0.24	
Ki-67			0.166
positive	21	0.69±0.56	
negative	18	0.46±0.39	
